# Advocacy for identifying certain animal diseases as “neglected”

**DOI:** 10.1371/journal.pntd.0005843

**Published:** 2017-09-07

**Authors:** François Louis Roger, Philippe Solano, Jérémy Bouyer, Vincent Porphyre, David Berthier, Marisa Peyre, Pascal Bonnet

**Affiliations:** 1 CIRAD, UMR ASTRE, Montpellier, France; 2 IRD, UMR INTERTRYP, Montpellier, France; 3 CIRAD, UMR SELMET, Montpellier, France; 4 CIRAD, UMR INTERTRYP, Montpellier, France; 5 CIRAD, ES Department, Montpellier, France; University of Minnesota, UNITED STATES

According to the World Health Organization (WHO), neglected tropical diseases (NTDs) affect almost 1,000,000,000 people in 149 countries (http://www.who.int/neglected_diseases/diseases/en/). Most are small family farmers living in the least-developed countries where health systems are often inadequate, and climate conditions are favourable to infectious and parasitic diseases. These diseases hinder socioeconomic development, maintain poverty, and impede the achievement of UN sustainable development goals (SDGs) [1]. Economic analyses have shown that their control, elimination, or eradication would lead to net economic benefits [2]. Rapid progress can be achieved when organised health systems, whether public, private, or mixed, are in place to provide diagnostic methods and facilities, treatments, and vaccines. Of the 18 diseases on WHO’s list of NTDs, only 5 are zoonoses: *Taenia solium* cysticercosis, echinococcosis, leishmaniasis, rabies, and human African trypanosomiasis (HAT). Some authors consider this list incomplete and believe that at least 3 other major zoonoses—anthrax, bovine tuberculosis, and brucellosis—should be included [3]. Moreover, while some public–private partnerships—e.g., the Global Alliance for Livestock Veterinary Medicines, GALVmed (https://www.galvmed.org/)—now target major livestock diseases impacting smallholders by connecting academia, public research institutes, and the pharmaceutical sector, no strictly animal disease (i.e., nonzoonotic) has been labelled “neglected”. Nevertheless, animal diseases directly impact people’s livelihoods. Furthermore, due to the multiple functions fulfilled by livestock in rural societies—as sources of food, income, and social status—animal diseases ultimately also impact human health.

## Observed impacts of labeling zoonotic diseases as “neglected”

The authoritative neglected status of the zoonoses on the WHO list of NTDs has allowed huge donations of drugs by pharmaceutical companies and financial donations by governments and philanthropic foundations. It also has facilitated the progress and funding of research, mobilised stakeholders, and helped curb the collapse of expertise.

HAT, or sleeping sickness, is a good example. Caused by trypanosomes transmitted by tsetse flies, HAT can cause death if not treated; there is no vaccine, no chemoprophylaxis, and no treatments that can be massively administered. After being neglected for decades, the growing numbers of victims (300,000 people infected in the early 1990s) and people exposed (60 million in Africa) finally prompted a coordinated response. WHO mobilised senior government officials, donors, research centres, and pharmaceutical companies, which committed to provide WHO drugs free of charge for distribution. Stakeholders reorganised their operations. This mobilisation ultimately reversed the disease’s upward trend, and, in 2015, fewer than 3,000 cases were reported. The success led WHO to include HAT in its NTD Roadmap, with the goal of eliminating the disease by 2030. Several donors (e.g., B&M Gates Foundation, Belgian government) have committed their support.

Rabies, which persists in tropical zones despite the existence of effective means of intervention, is another example. Pilot studies have demonstrated the feasibility of eradicating this disease at local and regional levels [4]. Research findings are informing advocacy work, which is increasing, leading the media and public authorities to pay rabies greater attention. In 2015, OIE (World Organisation for Animal Health), FAO (Food and Agriculture Organisation of the United Nations), and WHO called for massive investment to eradicate rabies at the global level (http://www.oie.int/en/animal-health-in-the-world/rabies-portal/).

A third example is *Taenia solium* cysticercosis, which, in low-income countries, is both an endemic health issue for local populations and a heavy economic burden for pig farmers [5]. Recent biomedical and epidemiological advances are driving international agencies, governments, and decision-makers to develop ad hoc management measures despite the remaining challenges in terms of setting up suitable surveillance and control measures [6].

## From zoonoses to animal diseases

There are 2 main reasons why we believe certain animal diseases should be labelled as “neglected”. First, as demonstrated by the cases on WHO’s list of NTDs, this label helps to attract, pool, and sustain funding and coordinate disease-control efforts. It also boosts research and operations in the field and helps to mobilise the private sector to manufacture and distribute drugs and vaccines. Second, animal diseases impact households through livestock mortality, decreased production or product quality (meat, milk, leather), and disorganised value chains and agroindustries, resulting in income losses [7] that, in turn, affect food security and human health. The risks posed by these diseases prevent livestock farmers from investing, trapping them in poverty. Such diseases—e.g., Newcastle disease, African swine fever, helminthiases, etc.—can even impact the national economies of countries that rely heavily on agriculture and animal production. More broadly, like NTDs, animal diseases can have an impact on most SDGs [1].

African animal trypanosomosis (AAT) illustrates our point. Like HAT, AAT is a trypanosomosis mainly transmitted by tsetse flies. Yet, in contrast with HAT, AAT is not listed as an NTD despite its major impact on livestock farming [8] and agricultural development in Africa [9]. With minimal additional investment, efforts currently underway to combat HAT could also focus on eliminating AAT in areas in which both forms of the disease co-occur [10]. This is the case, for example, in Chad, Central African Republic, Sudan, Cameroon, and Côte d’Ivoire. Protecting cattle from AAT also helps to protect humans from HAT [11]. The launching of the Pan-African Tsetse and Trypanosomiasis Eradication Campaign (PATTEC) by the African Union in 2000 demonstrates the political willingness of African leaders to rid the most vulnerable populations of this double burden of human and animal trypanosomoses. Contagious bovine pleuropneumonia (CBPP), endemic in sub-Saharan Africa, is another example of an animal disease that has been largely overlooked to date despite its significant socioeconomic impact [12]. This has resulted in weak surveillance, prevention (biosecurity measures, restrictions on cattle movements), vaccination, and treatment measures. As a “disregarded” disease, CBPP has entered a vicious circle in which a lack of sanitary data and economic information has resulted in a lack of interest on the part of donors and researchers, contributing to the implementation of inappropriate policies and measures, which in turn make it impossible to gather reliable data. Lumpy skin disease, which has been endemic in sub-Saharan Africa for decades and is now emerging in parts of the Middle East, the Caucasus, and southeastern Europe (Balkan Peninsula), is yet another example. In less-developed countries, it continues to cause substantial economic losses in terms of meat and milk production, major losses to the leather industry, and can result in regional or international trade restrictions [13].

## The way forward

“One Health” epidemiological research could help to better quantify the indirect role of animal diseases on human health by measuring the association between exposure (the occurrence of animal diseases) and outcomes (both human health indicators and social and economic impacts).

Economic evaluations should be used more extensively to identify the priority animal diseases to be tackled. For example, the cost-effectiveness or benefit ratios of disease-control measures in the field and avoided losses can be compared. More broadly, studies looking at prioritisation criteria and methods may be used to select the animal diseases to be identified and then recognised as “neglected”.

However, more transparent, standardised and reproducible methods, using objective criteria [14] and social and economic assessment approaches, are also needed. The first step would be to better describe and understand the impact of animal diseases on livelihood systems, i.e., on the activities of socially, economically, or geographically marginalised populations [15]. This impact must be evaluated first in terms of its pathway, meaning the mechanisms by which the disease affects agricultural household livelihoods, and then by the obstacles it represents to intensification and modernisation of farms [7], which are keeping households below poverty line. Finally, the impact may be assessed by studying spillover mechanisms hampering the functioning of other economic agents in markets and supply chains, thereby leading to greater food insecurity (lower prices, trade embargoes). In contrast with those used for human and zoonotic diseases, these criteria are more economic and social in nature.

Existing criteria in public health could be tailored to the animal world, such as the Disability Adjusted Life Years (DALY) synthetic indicator [16]. DALY indicators solely measure the burden of human diseases and are calculated based on the number of years lost due to a disability linked to poor health and to premature mortality due to disease. Moreover, at the community level, participatory methods could be used to involve smallholders in determining disease priorities and to understand their perception of sanitary risks.

## Conclusion

No strictly animal (nonzoonotic) disease has ever been labelled as “neglected” by international organisations, mainly because of the scarcity of evidence and the effort needed to transfer knowledge from science to policy-makers in the least developed countries. However, some animal diseases have a huge impact on the more vulnerable populations, especially in less developed areas of low and middle-income countries. These vulnerable populations usually suffer the double burden of “human” NTDs and animal diseases. Additional potential benefits stemming from an official recognition of neglected animal diseases are worth highlighting. For example, the recognition of ill-managed infectious (e.g., CBPP) and parasitic diseases and vectors could contribute to reducing the massive misuse of drugs and, therefore, better manage resistance to antimicrobials (AMR), antiparasitics, and insecticides. Official recognition of the most severe animal diseases’ neglected status could help lobbying and advocacy efforts to reduce the burden of these diseases both at the household and country levels. Furthermore, as underlined by the private sector (Pers. Com., P-M Borne, CEVA): "by nature, most of the neglected diseases don't attract enough private entities to invest sufficiently in research and development and to set up a sustainable value chain allowing an access to the ultimate beneficiaries”.

We therefore encourage international agencies and diverse stakeholders to consider the interest of tagging some animal diseases as “neglected”.
